# Dataset on large area nano-crystalline graphite film (NCG) grown on SiO_2_ using plasma-enhanced chemical vapour deposition

**DOI:** 10.1016/j.dib.2019.103923

**Published:** 2019-04-16

**Authors:** Camelia Albu, Sandra A.V. Eremia, Monica Lucia Veca, Andrei Avram, Radu Cristian Popa, Cristina Pachiu, Cosmin Romanitan, Mihaela Kusko, Raluca Gavrila, Antonio Radoi

**Affiliations:** a Centre of Bioanalysis, National Institute of Research and Development for Biological Sciences – Bucharest, 296 Splaiul Independentei, Bucharest, 060031, Romania; b National Institute for Research and Development in Microtechnology – IMT Bucharest, 126A Erou Iancu Nicolae Street, 077190, Voluntari, Romania

**Keywords:** Plasma-enhanced chemical vapour deposition (PECVD), Nano-crystalline graphite (NCG), Electrochemistry, Caffeic acid

## Abstract

A Si wafer coated with a low temperature oxide (LTO) was used as substrate (Si/SiO_2_) during the deposition of a thick nano-crystalline graphite (NCG) film by means of plasma-enhanced chemical vapour deposition (PECVD) procedure. The process parameters, the atomic force (AFM) and scanning electron (SEM) micrographs, Raman spectrum and X-ray diffraction (XRD) pattern are herein illustrated. The as deposited NCG film was electrochemically pretreated (3 mA applied current, during 240 s, in 10 mM phosphate buffer saline (PBS) solution containing 0.1 M KCl, pH 7) and thereafter used as electrode for sensing the caffeic acid content in lyophilised berries and dried chokeberries in “Nano-crystalline graphite film on SiO_2_: Electrochemistry and electro-analytical application” [1].

**Table:** Specifications table

Subject area	Electrochemistry, Materials science, Nanotechnology
More specific subject area	Electrode material development
Type of data	Image (atomic force microscopy – AFM, scanning electron microscopy – SEM), graph (Raman spectroscopy, X-ray diffraction – XRD), table (process parameters for using plasma-enhanced chemical vapour deposition – PECVD).
How data was acquired	Atomic force microscope (Ntegra Aura Scanning Probe Microscope, NT_MDT Spectrum Instruments) operated in intermittent-contact mode; scanning electron microscope (Nova NanoSEM 630, FEI Company, USA) working at 20 kV accelerating voltage, under high vacuum (HV) conditions and using Through the Lens Detector (TLD); confocal microscope (WITec alpha300 S, WITec, GmbH, Germany) with a 20X lens and 532 nm excitation wavelength was used to acquire the Raman spectrum; 9kW rotating anode X-ray diffraction system (Rigaku SmartLab, Japan) that employs Cu Kα1 radiation (λ = 1.54056 Å); PECVD growth (NANOFAB 1000, Oxford Instruments, UK).
Data format	Raw, analyzed
Experimental factors	Electrochemical activation of the nano-crystalline graphite film at 3 mA applied current, during 240 s, in 10 mM phosphate buffer saline (PBS) solution containing 0.1 M KCl, pH 7.
Experimental features	A 4″, Si-n, <100>, 1–3 Ω cm wafer bearing 110 nm SiO_2_ was heated up to 900 °C (15 °C min^−1^) in Ar/H_2_ (5%) atmosphere, annealed during 10 minutes, allowing an additional surface hydrogenation step for 5 more minutes in Ar/H_2_ (10%) atmosphere, after the ultimate process temperature was reached. The nano-crystalline graphite (NCG) film was grown starting from CH_4_ and H_2_ (60 sccm/75 sccm), using 100 W power plasma, at 900 °C and 1.5 Torr, up to a thickness of ∼350 nm. The working parameters of the PECVD process are detailed in Table 1. The NCG film grew on Si/SiO_2_ substrate was used as working electrode during several electrochemical investigations, alongside with Ag/AgCl reference electrode and Pt wire serving as counter electrode.
Data source location	National Institute for Research and Development in Microtechnology – IMT Bucharest, 126A Erou Iancu Nicolae Street, 077190, Voluntari, Ilfov county, Romania
*Data accessibility*	The data presented in this article are accessible within this article.
Related research article	C. Albu, S.A.V. Eremia, M.L. Veca, A. Avram, R.C. Popa, C. Pachiu, C. Romanitan, M. Kusko, R. Gavrila, A. Radoi, Nano-crystalline graphite film on SiO_2_: Electrochemistry and electro-analytical application, Electrochim. Acta, 303, 2019, 284–292 [1].

**Table:** 

**Value of the data** • The data are useful to understand the experiments performed in Ref. [1]. • The data are useful to researchers working in the field of electrochemistry, materials science and nanotechnology. • The data are relevant for nano-crystalline graphite (NCG) film growth through plasma-enhanced chemical vapour deposition (PECVD).

## 1 Data

**Fig. 1:**
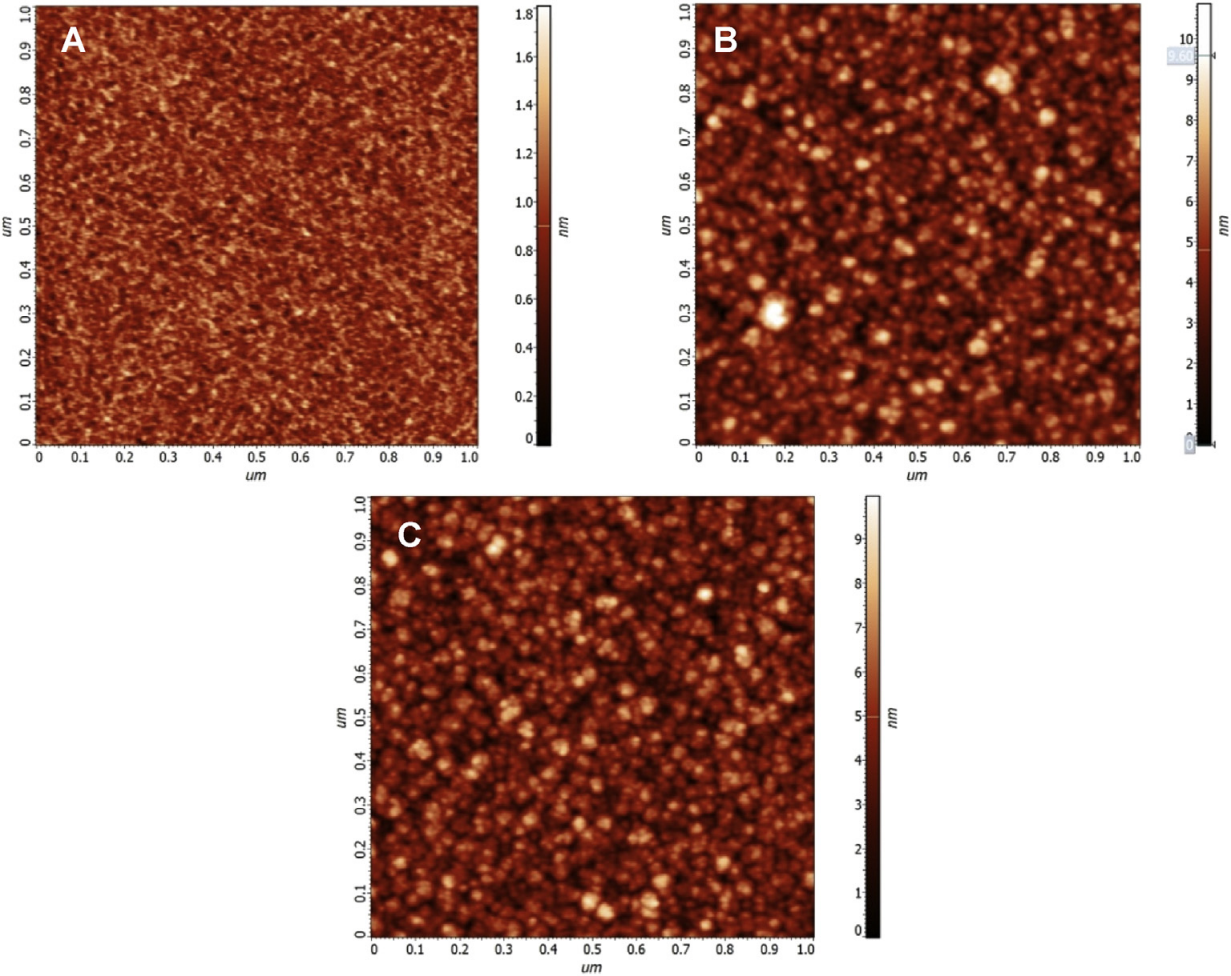
AFM illustrating (A) Si substrate- RMS 0.15 nm; (B) Si/LTO substrate– RMS 1.4 nm; and (C) Si/LTO –annealed substrate– RMS – 1.3 nm.

**Fig. 2:**
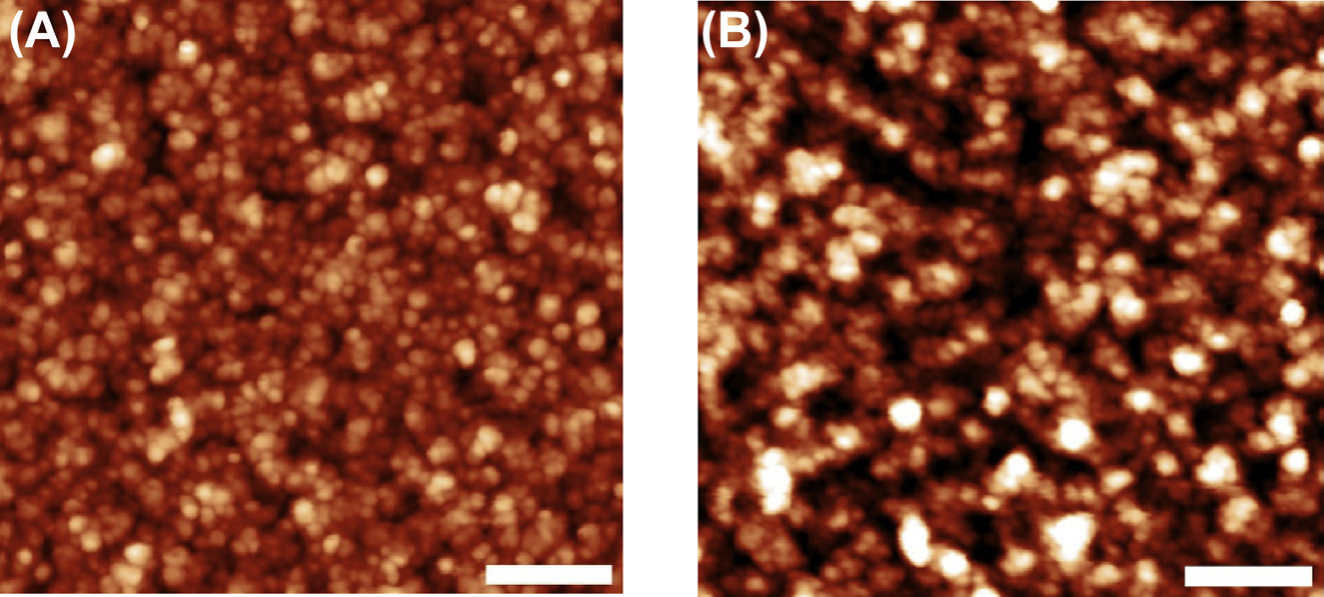
Atomic force microscopy (AFM) imaging before (A) and after (B) the electrochemical activation (scale bar: 200 nm).

**Fig. 3:**
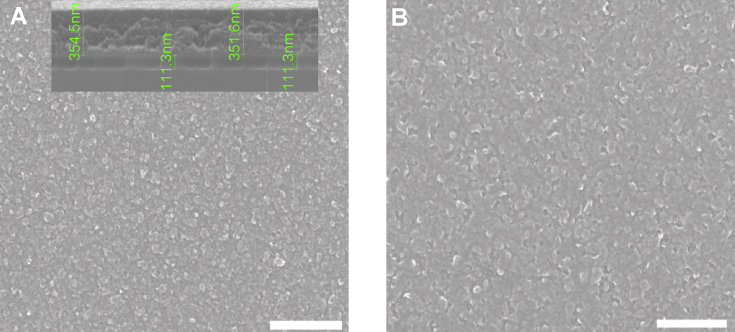
SEM micrographs before (A) and after the electrochemical activation (B) (scale bar: 300 nm).

**Fig. 4:**
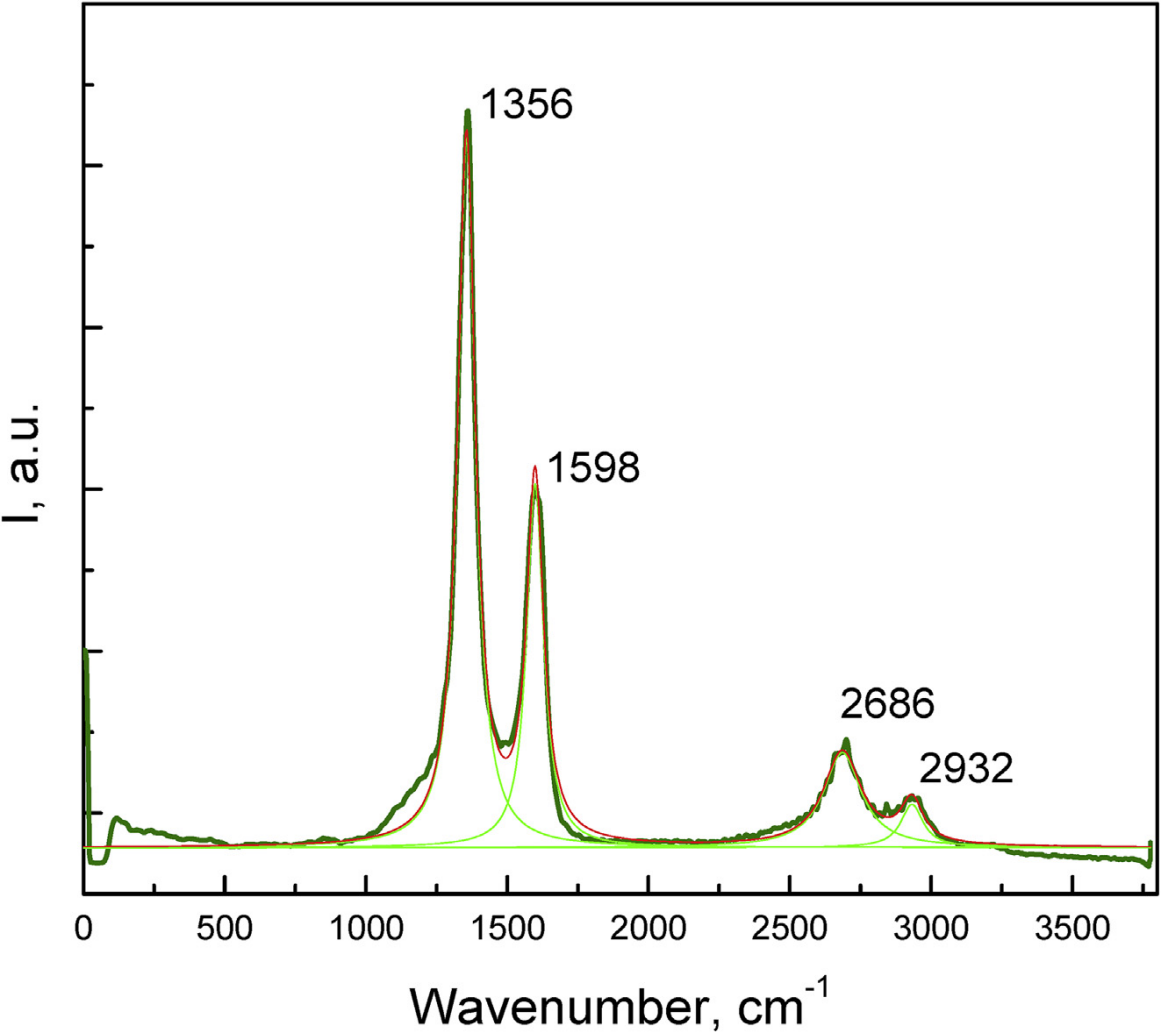
Raman spectra acquired at 532 nm; Lorentzian multiple peak fitting was used.

**Fig. 5:**
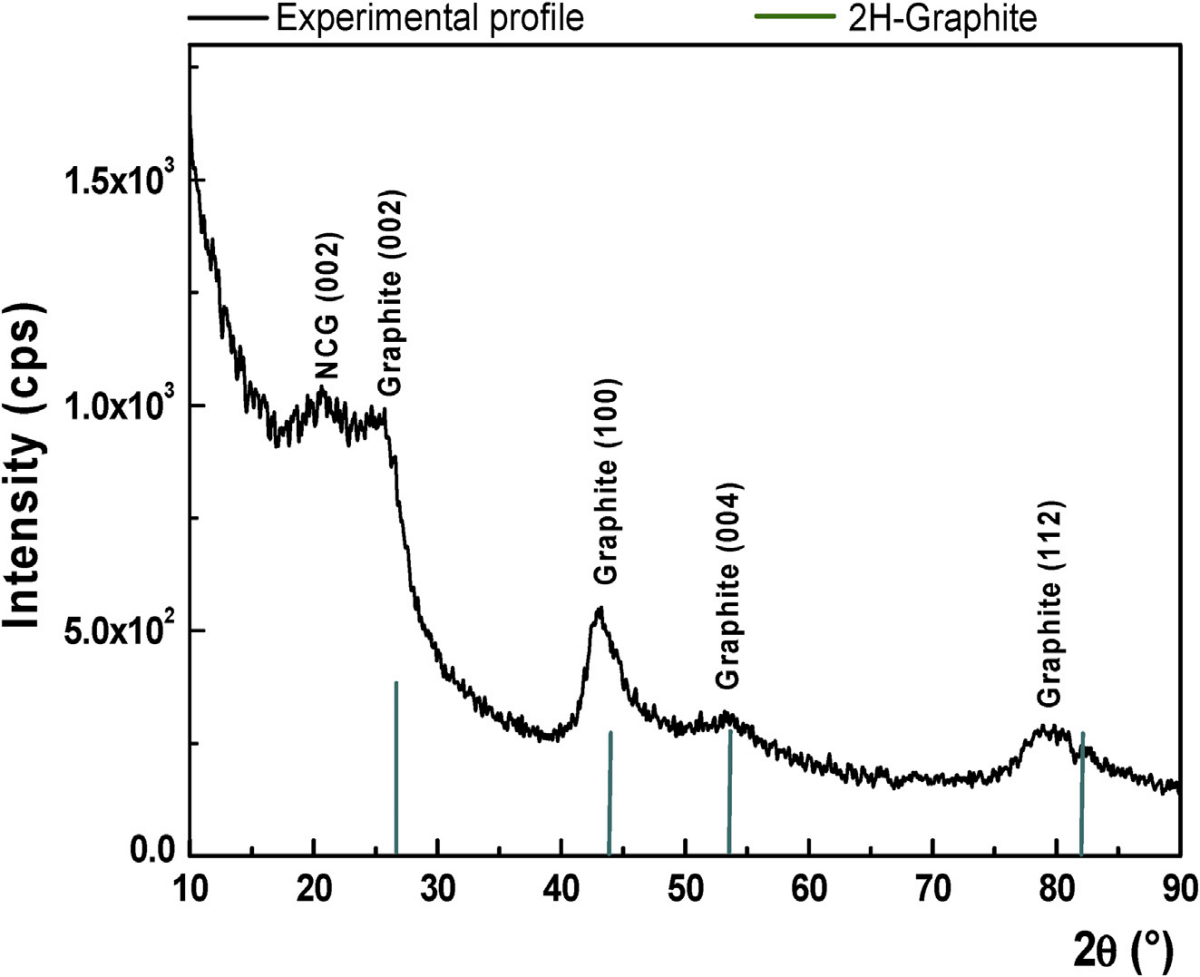
X-ray diffraction (XRD) pattern for nano-crystalline graphite (NCG) film.

The data are Supplementary materials paired with “Nano-crystalline graphite film on SiO_2_: electrochemistry and electro-analytical application” [1]. AFM micrographs in Fig. 1 are illustrating the Si substrate- RMS 0.15 nm (A), the Si/LTO substrate– RMS 1.4 nm (B) and the Si/LTO –annealed substrate– RMS – 1.3 nm (C). Fig. 2 shows typical morphologies, acquired using atomic force microscopy (AFM), of the NCG film before (A) and after the electrochemical pretreatment (B). Fig. 3 reports SEM micrographs before (A) and after the electrochemical activation (B). The thickness for the NCG film (∼350 nm) and the LTO deposited SiO_2_ (110 nm) is illustrated in Fig. 3A – inset. Fig. 4 exemplify the obtained Raman spectra acquired at 532 nm, using the confocal microscope WITec alpha300 S. X-ray diffraction pattern for nano-crystalline graphite (NCG) film is reported in Fig. 5. Table 1 provides the process parameters for LTO annealing and NCG growth achieved using the PECVD approach.

**Table 1:** Process parameters for LTO annealing and NCG growth.

	T, (°C)	t, (min)	Heat ramp (°C/min)	PRF., (W)	P, (mTorr)	Ar flow (sccm)	H_2_ flow (sccm)	CH_4_ flow (sccm)
Heat-up	200↗900	–	15	–	3000	1500	75	–
Si/SiO2 (LTO) annealing	900	10	–	–	3000	1500	75	
Hydrogen annealing	900	5	–	–	1500	1500	200	–
Deposition	900	120	–	100	1500	–	75	60
Cool-down	900↘200	–	9	–	1500	1500	–	–

## 2 Experimental design, materials and methods

Atomic force microscopy scans on bare Si wafer, Si/LTO –annealed substrate, as PECVD deposited nano-crystalline graphite film (NCG) and electrochemically etched NCG film, have been performed in intermittent-contact mode, using AFM probes provided with high aspect ratio tips (7:1, Olympus OMCL-AC240BSA) having 5–10 nm nominal radius. The micrographs were obtained using the Nova NanoSEM 630 (FEI Company, USA) scanning electron microscope (SEM). The Raman spectrum was acquired at 532 nm excitation wavelength using a with a WITec alpha300 S (WITec, GmbH, Germany) confocal microscope with a 20X lens; calibration was performed on the 520 nm Raman shift of Si. X-ray diffraction pattern of the NCG layer was obtained using a 9kW rotating anode (Rigaku SmartLab, Japan) that employs Cu Kα1 radiation (λ = 1.54056 Å). The electrochemical treatment (3 mA applied current, during 240 s) of the NCG film was performed using the Autolab electrochemical system model PGSTAT 302 N (Eco Chemie, The Netherlands) and a flat cell from Bio-Logic SAS (http://www.bio-logic.net/) in a three-electrode configuration, i.e. the working electrode being the NCG layer, the reference electrode was Ag/AgCl and as counter electrode a Pt wire was used. The electrolyte consisted of 10 mM phosphate buffer saline (PBS) solution, pH 7, supplemented with 0.1 M KCl. A Si/SiO_2_ substrate (4″, Si-n, <100>, 1–3 Ω cm wafer with 110 nm low temperature oxide) was heated up to 900 °C (15 °C min^−1^) in Ar/H_2_ (5%) atmosphere, annealed during 10 minutes, additionally treated 5 more minutes in Ar/H_2_ (10%) atmosphere, after reaching the ultimate process temperature. The nano-crystalline graphite (NCG) film was grown starting from CH_4_ and H_2_ (60 sccm/75 sccm), using 100 W power plasma, at 900 °C and 1.5 Torr, up to a thickness of ∼350 nm.
