# Supplementary material for: Dataset on large area nano-crystalline graphite film (NCG) grown on SiO2 using plasma-enhanced chemical vapour deposition
Source: Data Brief. 2019 Apr 16;24:103923. doi: 10.1016/j.dib.2019.103923 (PMC6484281; doi:10.1016/j.dib.2019.103923)
Supplement: Multimedia component 1 [file mmc1.pdf]

Declaration of conflict of interest

I hereby declare, on behalf of all co-authors, that no conflict of interest is competing in between all the authors! I declare that the data submitted with this paper was not published elsewhere nor is submitted for publication!

Best regards,

Sandra Eremia
